# Development of Highly Sensitive Fluorescent Sensors for Separation-Free Detection and Quantitation Systems of Pepsin Enzyme Applying a Structure-Guided Approach

**DOI:** 10.3390/bios14030151

**Published:** 2024-03-20

**Authors:** Aya M. Mostafa, Stephen J. Barton, Stephen P. Wren, James Barker

**Affiliations:** 1 School of Life Sciences, Pharmacy and Chemistry, Kingston University, Kingston upon Thames, London KT1 2EE, UK; s.barton@kingston.ac.uk (S.J.B.); s.wren@kingston.ac.uk (S.P.W.); j.barker@kingston.ac.uk (J.B.); 2 Department of Pharmaceutical Analytical Chemistry, Faculty of Pharmacy, Assiut University, Assiut 71526, Egypt

**Keywords:** fluorescent molecularly imprinted polymers, fluorescent biosensors, spectrofluorometric analysis, pepsin, biomarkers, gastroesophageal reflux disease (GERD)

## Abstract

Two fluorescent molecularly imprinted polymers (MIPs) were developed for pepsin enzyme utilising fluorescein and rhodamine b. The main difference between both dyes is the presence of two (diethylamino) groups in the structure of rhodamine b. Consequently, we wanted to investigate the effect of these functional groups on the selectivity and sensitivity of the resulting MIPs. Therefore, two silica-based MIPs for pepsin enzyme were developed using 3-aminopropyltriethoxysilane as a functional monomer and tetraethyl orthosilicate as a crosslinker to achieve a one-pot synthesis. Results of our study revealed that rhodamine b dyed MIPs (RMIPs) showed stronger binding, indicated by a higher binding capacity value of 256 mg g^−1^ compared to 217 mg g^−1^ for fluorescein dyed MIPs (FMIPs). Moreover, RMIPs showed superior sensitivity in the detection and quantitation of pepsin with a linear range from 0.28 to 42.85 µmol L^−1^ and a limit of detection (LOD) as low as 0.11 µmol L^−1^. In contrast, FMIPs covered a narrower range from 0.71 to 35.71 µmol L^−1^, and the LOD value reached 0.34 µmol L^−1^, which is three times less sensitive than RMIPs. Finally, the developed FMIPs and RMIPs were applied to a separation-free quantification system for pepsin in saliva samples without interference from any cross-reactors.

## 1. Introduction

Molecular imprinting technology is an innovative technique that has provided synthetic and highly selective receptors for different target analytes. Molecular imprinting is a simple technique that depends on the copolymerisation of functional monomer(s) and crosslinker(s) in the presence of the target analyte as a template. Herein, non-covalent bonds are mainly established between the template and the functional monomer that are the bases for molecular identification [1]. Upon polymerisation, complementary binding sites are created for the target analyte in the structure of the polymer, which, after removing the template using a suitable solvent, become available for binding this analyte in different samples [2,3,4,5]. The growing interest in molecular imprinting technology is due to the increased advantages it offers in comparison to their biological alternative, antibodies. Up till the present, antibodies have been used for the detection of different biological analytes, specifically through enzyme-linked immunosorbent assay (ELISA), due to their extremely high selectivity. However, the sensitivity of antibodies to heat and pH, their high cost of production, and their short shelf life are significant drawbacks. Moreover, some antibodies need to be labelled for the detection of the binding event, which is not a straightforward procedure and may result in an alteration of the binding properties of the antibodies. Therefore, molecular imprinting technology proved to be an ideal alternative for antibodies as molecularly imprinted polymers (MIPs) are more stable, cost-effective, more resistant to degradation, and can be tailor-made to bind any analyte of interest [6].

Labelling of MIPs with a fluorescent reporter can be a very useful tool that helps to translate the binding event between MIPs and the target into a readable signal. Creating fluorescent MIPs can be achieved through three major approaches: (1) incorporating fluorescent particles into the core of the developed polymer, such as carbon dots [7,8], quantum dots [9,10,11,12], or gold nanoparticles [13] in a technique known as core-shell imprinting; (2) the use of a fluorescent functional monomer in the polymerisation mixture [14,15,16]; or (3) post imprinting modification of the resulting MIPs with a fluorescent dye [17]. Nonetheless, the first two approaches are more popular and involve less risk of alteration of the binding properties of the developed MIPs [18]. Fluorescent MIPs not only allow readable detection of the binding event but also the exploitation of the fluorescence as a quantitation method for the concentration of the target. In this case, the binding of the target to fluorescent MIPs results in an alteration of the fluorescence, either an increase (less common) or a decrease in the fluorescence intensity, which is usually proportional to the concentration of the analyte. Therefore, fluorescent MIPs provide not only a method for extraction and detection of the target analyte but also a very sensitive and accurate method of quantitation, which makes them a very inclusive and self-sufficient technique of analysis [19].

Fluorescein is a widely known fluorescent dye that belongs to the xanthene class of dyes, which has been used for decades in fluorescent labelling. The incorporation of fluorescein into the structure of MIPs has been successfully reported multiple times to determine different targets, owing to its strong fluorescence and market availability [20]. In the paper reported by F. Wang et al., fluorescein has been applied in the development of fluorescent MIPs for the detection of naproxen, a common non-steroidal anti-inflammatory drug, through a simple and catalyst-free sol-gel polymerisation method [21]. The authors acknowledged that the achieved limit of detection for the developed MIPs was not satisfactory in comparison to other methods reported for naproxen, and they suggested the use of a more sensitive dye. Therefore, in light of this recommendation, we present the development of two fluorescent MIPs utilising fluorescein and rhodamine b as fluorescent dyes for the detection of the pepsin enzyme. Fluorescein and rhodamine b are conjugated through a simple reaction with (3-aminopropyl)trimethoxysilane to create the fluorescent co-monomer. This fluorescent co-monomer is incorporated in a one-pot sol-gel polymerisation method to produce the yellow-coloured FMIPs from fluorescein and the pink-coloured RMIPs from rhodamine b.

Pepsin was chosen as a target for our work because it has been recently confirmed as a biomarker for gastroesophageal reflux disease (GERD). GERD is a common disorder occurring in over 20% of the world’s population [22]. The early diagnosis and treatment of GERD can efficiently contribute to the prognosis of this common disease. The pepsin enzyme is one of the earliest discovered enzymes that aid in the digestion of proteins in humans and animals. However, the enzyme can also cause increased damage to the mucosal lining of the stomach in patients with GERD, resulting in more pain [23]. Furthermore, by analysing the saliva of patients with GERD, it was found that the saliva exhibited higher levels of pepsin enzyme compared to healthy individuals. As a result, elevated levels of pepsin in the saliva of patients could be used as a biomarker for GERD and act as an early, simple, and non-invasive diagnostic tool [24]. Up till now, the main method of measuring the level of pepsin and diagnosis of GERD is the Peptest^®^, which is an ELISA method utilising antibodies [25]. However, we aim to find a synthetic, cheaper, and more stable alternative to conventional antibody testing. 

Our group has recently focused on the synthesis of MIPs as extraction and/or analytical tools for pepsin as an important biomarker for GERD. Imprinting of proteins can be challenging due to their big size, pH sensitivity, conformational stability, and limited solubility [26]. Nonetheless, there has been a huge progress in the field of imprinting of proteins through the use of more targeted monomers, suitable solvents and epitope imprinting [27]. In our review of the literature, very few papers dealt with the imprinting of pepsin [28,29], of which only one paper described the development of fluorescent MIPs for the detection of pepsin in an ELISA-like format [30]. In this reported study, the analysis of pepsin was based on the binding of fluorescent pepsin-specific MIPs to magnetic pepsin nanoparticles immobilised on magnetic inserts in the wells of a microtiter plate. Herein, free pepsin in the sample competed with immobilised pepsin, causing a decrease in fluorescent MIPs binding to the magnetic inserts, hence increasing the central fluorescence of the well. The synthesis of fluorescent MIPs in this reported work was not straightforward and included multiple steps. There was no optimisation of the concentration or types of monomers or crosslinkers and no justification for the choice of monomers applied in their method. In addition, no chemical, thermal, or functional characterisation profiles, including kinetics and isotherm, were provided. Finally, there was no application on human saliva samples. In one of our previous works, we developed magnetic MIPs for the extraction of pepsin, utilising the core-shell imprinting technique [31]. The developed magnetic MIP bound pepsin in saliva samples, which was then released from MIPs and analysed via high-performance liquid chromatography. However, we aspired to design fluorescent MIPs that would be capable of both extraction and analysis of pepsin in a single step. Therefore, in this current work, we developed two fluorescent MIPs using fluorescein and rhodamine b as fluorescent reporters with complete optimisation and characterisation profiles. In addition, analytical method optimisation and validation were investigated for the determination of pepsin using both MIPs to compare their analytical performance. Finally, the fluorescent MIPs were used to demonstrate their applicability for the extraction and quantitation of pepsin in human saliva samples. 

## 2. Experimental Section

### 2.1. Materials and Instrumentation

Tetraethyl orthosilicate for synthesis (TEOS), sodium dodecyl sulphate 99% (SDS), (3-aminopropyl)triethoxysilane 98% (APTES), deionised water, absolute ethanol, sodium chloride 99.5%, phosphate-buffered saline (PBS) tablets (pH 7.2), human pepsin, human lipase, and human amylase were all procured from Thermo Fisher Scientific (Horsham, UK). Fluorescein-5-isothiocyanate 98% (FITC) and rhodamine b isothiocyanate 98% (RITC) were purchased from Fluorochem, (Hadfield, Glossop, UK). 

Fluorescence measurements were carried out using a Cary Eclipse fluorescence spectrometer (Agilent, London, UK), running on a Cary Eclipse software version 1.1 (132) (Agilent, London, UK). UV measurements were conducted using a Cary UV-Vis Compact, operating on a Cary UV Workstation™, software version 1.0.1284 (Agilent, London, UK). Thermal characterisation, including thermogravimetric experiments, was performed on a Mettler Toledo TGA/DSC 1 Series (Leicester, UK)—running on STARe™ software Version 10.00 (Mettler Toledo, Leicester, UK), and differential scanning calorimetric assays (DSC) were performed on a TA Instruments DSC25 Series (New Castle, UK), running on Trios™ software, v5.4.0.300 (TA instruments, New Castle, UK) Infra-red analysis was conducted using a Thermo Fisher (Horsham, UK) Scientific Nicolet iS5 Fourier transform infrared spectroscopy (FTIR) running on OMNIC™ software version 9.13.5.1294 (Thermofisher, Horsham, UK). ^13^C Nuclear magnetic resonance (NMR) analysis was carried out using a Bruker Avance III 600 two-channel FTNMR spectrometer (Coventry, UK) operating at 600 MHz and utilising TopSpin™ software version 4.3.0 (Bruker, Coventry, UK) for data analysis. Scanning electron microscopy (SEM) was used to visualise the resulting polymers using a Zeiss Evo-50 electron microscope (Cambridge, UK) operating on Smart SEM™ software version 5.0 (Zeiss, Cambridge, UK). Data processing and graph plotting were executed using Origin™ 8.5 software (Origin Lab Corporation, North Hampton, NH, USA). 

### 2.2. Synthesis of Fluorescent Co-Monomers

Two fluorescent co-monomers were prepared independently from a simple coupling reaction between APTES and FITC or RITC according to a previously reported method [32]. Each dye (0.04 mmol) was dissolved in 8 mL absolute ethanol, and 0.04 mmol of APTES was added to the solution and continuously stirred for 24 h. The obtained product was used without further purification.

### 2.3. Preparation of Fluorescent MIPs for Pepsin

Synthesis of fluorescent MIPs was carried out through a simple sol-gel polymerisation reaction, which is an effective, simple and green method [4]. APTES was employed as a functional monomer, and TEOS as a crosslinker. The amounts and volumes of different reagents used in the synthesis procedure were carefully optimised to reach the best possible binding. The optimised procedure for fluorescein dyed MIPs (FMIPs) was as follows: 15 mg of the target analyte pepsin were dissolved in 8 mL PBS buffer solution (pH 7.2) followed by the addition of 0.50 mL APTES and stirring for half an hour for preassembly followed by addition of 9 mL of the fluorescent co-monomer (FITC-APTES) and stirring for another half hour. Finally, 0.9 mL of TEOS dissolved in 3.5 mL of absolute ethanol was added dropwise, and the reaction mixture was sealed under a nitrogen atmosphere and continuously stirred for 48 h. The optimised procedure for rhodamine b dyed MIPs (RMIPs) was almost the same with different optimum volumes. Pepsin (15 mg) was dissolved in 8 mL PBS buffer (pH 7.2), followed by the addition of 0.75 mL APTES and stirred for half an hour, followed by the addition of 8 mL of the fluorescent co-monomer (RITC-APTES) and stirring for another half hour. Finally, 0.9 mL of TEOS dissolved in 3.5 mL absolute ethanol was added dropwise, and the reaction mixture was sealed under a nitrogen atmosphere and continuously stirred for 48 h. Fluorescent non-imprinted polymers (NIPs) were prepared for both FMIPs and RMIPs without the addition of pepsin for comparison purposes. The resulting MIPs and NIPs were rinsed with deionised water and ethanol twice to remove remnants of starting materials and oligomers. Furthermore, to remove the target from the imprinted cavities, both fluorescent MIPs were washed with a solution of 1% *w*/*v* sodium dodecyl sulphate (SDS)/10% *v*/*v* acetic acid for 4 h, followed by washing with deionised water multiple times to eliminate remnants of the washing solution. UV spectrometry was used to ensure complete washing by testing fragments of the washing solution. The resulting polymers were dried under vacuum at 60 °C and then finely ground and stored in dark containers to prevent photobleaching.

### 2.4. Protein Binding Experiments

To assess the successful imprinting, individual binding assays were performed for all the developed polymers against a known concentration of pepsin. The binding experiments were carried out by incubating 50 mg of the developed polymers with 20 mL of 1 mg mL^−1^ pepsin solution for 2 h (for fluorescein-dyed polymers) and 1 h (for rhodamine b dye polymers). After shaking for the optimum binding time, solutions were centrifuged at 4500 rpm for 10 min, and the concentration of pepsin in the supernatant was measured by UV spectrometry against a blank of deionised water incubated with MIPs or NIPs for the same amount of time. To evaluate the binding capacity of MIPs and NIPs, the amount of pepsin adsorbed per gram of polymers was calculated using the following equation:

$$Q=\left(Ci-Ct\right)\cdot V/m$$

where, *Q* (mg g^−1^) is the quantity of pepsin in milligrams adsorbed per gram of polymer, *Ci* (mg mL^−1^) is the starting concentration of pepsin, *Ct* (mg mL^−1^) is the remaining concentration of pepsin after incubation time (*t*), *V* (mL) is the volume of pepsin solution, and *m* (g) is the mass of MIPs or NIPs applied in the experiment. All the experiments were conducted in triplicate to validate the precision of the results.

### 2.5. Characterisation of the Fluorescent Polymers

Characterisation of the developed fluorescent polymers is a very important step in comparing the properties of FMIPs and RMIPs, especially the different binding parameters. Therefore, the morphology of the developed polymers was assessed using SEM imaging, which also enabled an estimation of the particle size. ^13^C nuclear magnetic resonance (NMR) was conducted on FITC, RITC, FITC-APTES, and RITC-APTES to verify the formation of the thiourea linkage between FITC or RITC with APTES to form the fluorescent co-monomers. Fourier transform infrared spectra (4000–500 cm^−1^) were collected for FITC, RITC, FMIPs, FNIPs, RMIPs, and RNIPs to compare the prominent bands, ensure template removal, and verify the absence of any residual starting materials.

Thermogravimetric analysis (TGA) and differential scanning calorimetry (DSC) were performed on FITC, RITC, FMIPs, FNIPs, RMIPs, and RNIPs. Data provided by TGA and DSC can ensure complete polymerisation, detect any unreacted starting materials, and determine the content of adsorbed moisture. TGA was performed along a temperature range from 25 to 650 °C at a heating pace of 10 °C min^−1^ and a nitrogen gas flowing at a rate of 50 mL min^−1^, and DSC was run at a temperature range from 25 to 350 °C at a heating rate of 10 °C min^−1^.

To determine the order of binding kinetics, rebinding experiments were performed on the developed polymers at increasing time intervals, I.e., 50 mg of the fluorescent polymers were incubated with 20 mL of 1 mg mL^−1^ pepsin solution for 0, 1, 2, 4, 6, and 8 h. The quantity of pepsin adsorbed per gram (Q) for each time interval was plotted against time to determine the binding order kinetics.

Additionally, rebinding experiments were conducted on the developed polymers by applying different concentrations of pepsin to determine the binding isotherm model. Thus, 50 mg of the fluorescent polymers were incubated with 20 mL of pepsin solutions in the concentration range (0.2 to 1.5 mg mL^−1^) for 1 h (for RMIPs and RNIPs) and 2 h (for FMIPs and FNIPs). The quantity of pepsin adsorbed per gram (Q) for each concentration was plotted against concentration to depict the binding isotherm model.

To verify the binding selectivity of FMIPs and RMIPs, their binding was compared to the binding of their corresponding FNIPs and RNIPs, respectively. Subsequently, the imprinting factor (IF) was calculated by dividing the value of Q for MIPs by that of NIPs. In addition, since the target analyte in this work is salivary pepsin, it is only reasonable to test the selectivity of the developed fluorescent polymers against other proteins that can exist with pepsin in human saliva. Amylase and lipase, along with other enzymes, were selected to test the selectivity due to their abundance in saliva and possible cross-reactivity. Therefore, a similar binding assay for pepsin was performed on the competitor enzymes where 20 mL of 1 mg mL^−1^ solution of each enzyme was incubated with 50 mg of the fluorescent polymers for 1 h (for RMIPs and RNIPs) and 2 h (for FMIPs and FNIPs). The quantity bound per gram (Q) was calculated for each enzyme and compared to that of pepsin.

### 2.6. Stability Testing

The developed MIPs were tested for their stability and shelf life to verify their suitability for long-term use. The developed FMIPs and RMIPs were left for 0- 1-, 3-, and 6-month periods after preparation while being stored in dark containers. In addition, other batches of FMIPs and RMIPs were stored at 10, 25, 35 °C and 45 °C for 1 month period to test for stability against storage temperatures. After these storage periods, the binding capacity of pepsin was tested using the procedure for protein binding assay mentioned previously.

### 2.7. Fluorescence Measurements

The procedure for measuring the fluorescence intensity of the developed fluorescent polymers was carefully optimised to get the highest possible sensitivity for FMIPs and RMIPs. Consequently, a suspension of the fluorescent polymers in water (3 mg mL^−1^) was prepared, centrifuged to remove coagulated particles, and measured at an excitation wavelength (λ_exc_) of 471 nm for FMIPs and 546 nm for RMIPs at PMT voltage of 600 and slit width of 10 nm for both excitation and emission. In order to construct a calibration curve, different concentrations of pepsin were added to the suspension of polymers for the optimum binding time of 2 h or 1 h for FMIPs and RMIPs, respectively, with continuous shaking. Relative fluorescence intensity was measured for each sample and plotted against the concentration of pepsin to establish the linear range and deduct the regression equation.

### 2.8. Application to Measuring Pepsin in Human Saliva

This study received ethical consent from Kingston University Ethics Committee (Ethics Code 2895) and was conducted in concordance with the regulations of the UK Human Tissue Act (HTA) 2004. Saliva samples were collected, centrifuged at 4500 rpm for 30 min, and used immediately. 100 µL of saliva were spiked with increasing concentrations of pepsin in the concentration range (0–42.85 µmol L^−1^). One mL of the suspension of FMIPs or RMIPs (3 mg mL^−1^) was added to each sample and incubated for the optimum binding time. Each sample was measured in triplicate using a spectrofluorometer to ensure the precision of the results.

## 3. Results and Discussion

### 3.1. Preparation of FMIPs and RMIPs

**Figure 1:**
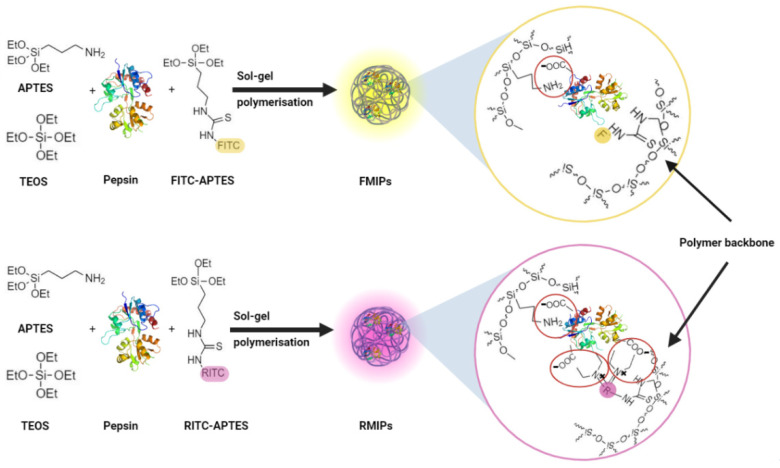
Schematic presentation of polymerisation and interaction points between pepsin and fluorescein or rhodamine b.

The use of fluorescein as a fluorescent dye for manufacturing of MIPs was previously reported for naproxen [20]. However, the authors of this paper stated that the use of fluorescein did not achieve the desired sensitivity levels and that other dyes needed to be tested. Based on this recommendation, we tested another cheap and readily available organic dye, rhodamine b, along with fluorescein. Although the structure of rhodamine b is very close to that of fluorescein, it still has some structural merit that we presumed can enhance binding and sensitivity. A neutral pH was chosen as the working pH since we detect pepsin in saliva, which has a neutral pH (6.2–7.6) [33]. As pictured in Figure 1, rhodamine b has two diethylamino groups in its structure, which are positively charged at the neutral working pH (7.2). Pepsin has a relatively low isoelectric point of 3.24 [34], which means that it is negatively charged at pH 7.2. Therefore, a strong electrostatic interaction was predicted between rhodamine b and pepsin, unlike fluorescein, which can only interact with pepsin through hydrogen bonds in its carboxylic group.

APTES was chosen as a functional monomer due to its ability to interact with the amino and carboxylic groups in the backbone of the pepsin molecule via hydrogen bonds. Moreover, APTES was also coupled to FITC or RITC to generate the fluorescent co-monomer, which is also capable of hydrogen bonding with the target. As a result, after washing the developed FMIPs or RMIPs and removal of the target, many binding sites complementary to pepsin would be generated. In addition, fluorescein or rhodamine b molecules would be distributed in the polymer matrix with high prevalence in the binding sites due to the precedent hydrogen and/or electrostatic bonds formed with the target during the polymerisation phase. Consequently, the binding and the release of pepsin to the binding sites would result in a significant change in the fluorescence intensity, which can be recorded and utilised to detect the presence of pepsin and determine its concentration. Additionally, a slight colour change between MIPs and their corresponding NIPs was noticed, in which MIPs always seemed darker in colour than NIPs, as shown in the pictures in Figure S1. This can be attributed to the greater concentration of the fluorescent co-monomer in the polymeric matrix of MIPs compared to NIPs; this observation lies in concordance with the same observations for fluorescein imprinted polymers previously prepared for naproxen [20]. Since the pepsin molecule can form multiple bonds with the fluorescent co-monomer, it is predicted that the presence of pepsin resulted in higher polymerisation efficiency of FITC-APTES or RITC-APTES into MIPs, resulting in a darker colour.

All the reagents used in the synthesis procedure were investigated thoroughly to study their effect on the binding capacity (Q). Different volumes of APTES, FITC-APTES or RITC-APTES, and TEOS were tested, as well as different preassembly times and different amounts of pepsin to fully optimise the procedure. Figures S2 and S3 show the optimum values obtained from the different optimisation experiments for both fluorescein-dyed polymers and rhodamine b-dyed polymers, respectively. In addition, testing different washing solutions such as sodium chloride (0.5 mol L^−1^), phosphate buffer (0.05 mol L^−1^, pH 7.2), and a solution of 1% *w*/*v* SDS/10% *v*/*v* acetic acid was necessary to find the solution that removes all traces of the template. This is very crucial as incomplete template removal results in template bleeding, which can cause significant error in the anticipated result and/or blockage of the available binding sites, leading to a reduction in sensitivity. Therefore, after meticulous testing of the different washing solutions and time of washing, a solution of 1% *w*/*v* SDS/10% *v*/*v* acetic acid was determined to be the most effective in the removal of the template within 4 h.

### 3.2. Characterisation of the Fluorescent MIPs

#### 3.2.1. Morphological Characterisation

**Figure 2:**
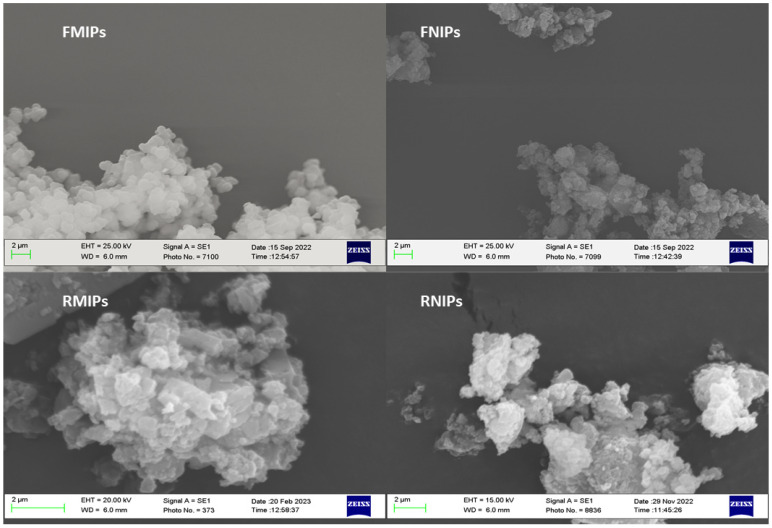
SEM pictures of FMIPs, FNIPs, RMIPs, and RNIPs.

SEM images were collected for FMIPs, FNIPs, RMIPs, and RNIPs and are displayed in Figure 2. As the images show, the generated polymers are not entirely spherical, with a rough surface and some scattered, coagulated portions. This is due to the application of the bulk polymerisation technique in which there is no complete control over the size or morphology of the synthesised polymers. Moreover, a basic estimation of the particle size was made using the resulting SEM pictures. Here, we can report that the particles have a size ranging from 0.5 to 2.0 microns, which again is typical for bulk polymerisation. 

To investigate the effect of the variability of particle size on fluorescence signal reproducibility, a one-way analysis of variance (ANOVA) test was conducted. One batch (3 mg mL^−1^) of each of the FMIPs, FNIPs, RMIPs, and RNIPs was prepared, and the fluorescence signal was measured three times for each solution. The same experiment was conducted every day over a week period. The collected data for seven days (seven groups) for each polymer type was tested via ANOVA analysis, and the results are displayed in Table 1. Since the F-statistic values are less than the critical values, they suggest that the differences between the group means are not statistically significant. This implies that there is no strong evidence to reject the null hypothesis, and we conclude that there are no statistically significant differences between the means of the groups for all polymers. In other words, the variation observed between the groups could likely be due to random chance alone, and there may not be real differences in the means of the groups.

#### 3.2.2. Chemical Characterisation 

**Figure 3:**
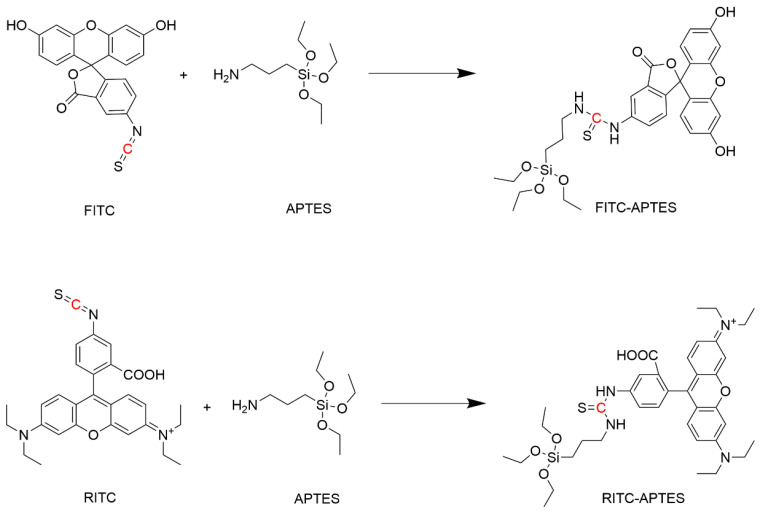
The reaction between FITC or RITC and APTES to form the fluorescent co-monomers.

To verify the formation of thiourea linkage between FITC or RITC with APTES, ^13^C NMR was conducted on the reactants and the products. The focus of this analysis was on the chemical shift of the carbon atom of the isothiocyanate group, which would convert to a thiourea bond after reaction with the amino group of APTES, as illustrated in Figure 3. Herein, we notice that the chemical shift value of the carbon atom of the isothiocyanate group of FITC changed from (δ = 138.2,s) to (δ = 180.7,s) due to the formation of thiourea with the amino group of APTES. Similar changes were observed for RITC, where the value of the chemical shift of the carbon atom of the isothiocyanate group changed from (δ = 137.9,s) to (179.3,s). NMR spectra for FITC and FITC-APTES, RITC and RITC-APTES are demonstrated in Figures S4 and S5, respectively.

**Figure 4:**
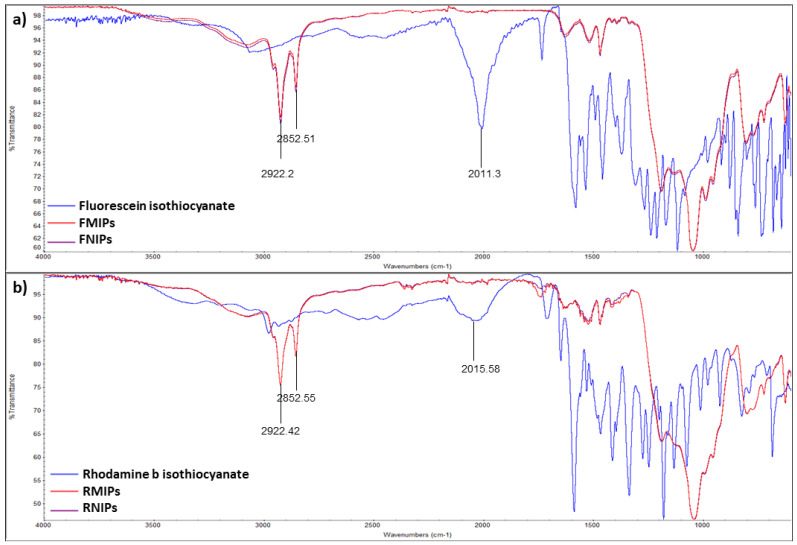
Infrared spectra for (**a**) FITC, FMIPs, and FNIPs and (**b**) RITC, RMIPs, and RNIPs.

The synthesised polymers, as well as the organic dyes, were characterised via IR spectroscopy to confirm the complete involvement of the fluorescent dyes in the developed polymers and the spectra are shown in Figure 4. Both FITC and RITC show a distinct peak at ~2010 cm^−1^, which is characteristic of the isothiocyanate group. As a result, we notice the disappearance of this peak in the resulting polymers’ spectra, which verifies the incorporation of the dye into the polymeric structure. In addition, we observe that FMIPs and FNIPs, as well as RMIPs and RNIPs, have almost identical spectra since there is no structural difference between them. There were no peaks in the range of 3000 to 3300 cm^−1^, characteristic of amino and carboxylic groups, which indicates the complete removal of pepsin from the binding sites of both MIPs.

#### 3.2.3. Thermal Characterisation 

**Figure 5:**
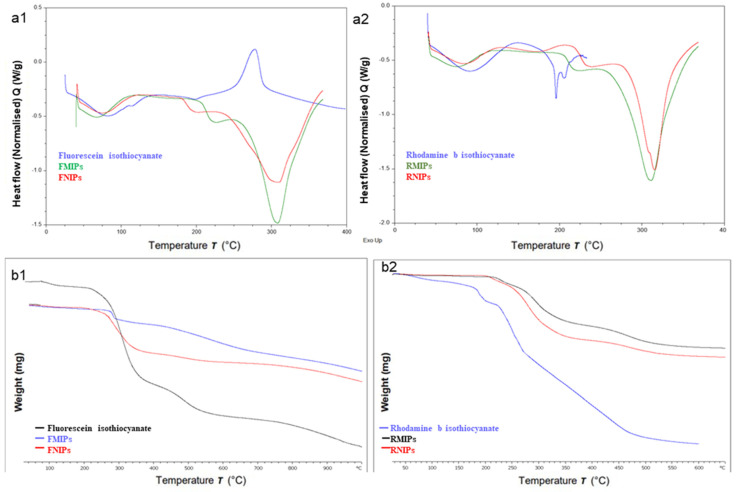
DSC thermograms for (**a1**) FITC, FMIPs, and FNIPs, (**a2**) RITC, RMIPs, and RNIPs and TGA graphs for (**b1**) FITC, FMIPs, and FNIPs, (**b2**) RITC, RMIPs, and RNIPs.

DSC thermograms (Figure 5(a1,a2)) collected for the organic dyes and their polymers provided further confirmation of the incorporation of the dyes in the structure of the developed polymers. The DSC curve of FITC shows only an exothermic peak around 290 °C, yet DSC curves of both FMIPs and FNIPs show only one melting peak at around 320 °C, indicating the absence of any residual FITC. Similarly, the DSC thermogram for RITC showed a melting peak of 210 °C. However, the DSC graphs of both RMIPs and RNIPs showed only one melting peak at 320 °C, which proves the absence of any residual fluorescent dye. This data was further confirmed with TGA (Figure 5(b1,b2)), which showed no notable difference in the decomposition pattern between FMIPs and FNIPs and RMIPs and RNIPs due to their structural similarity. However, their decomposition patterns are notably different from that of the fluorescent dyes.

#### 3.2.4. Functional Characterisation

**Figure 6:**
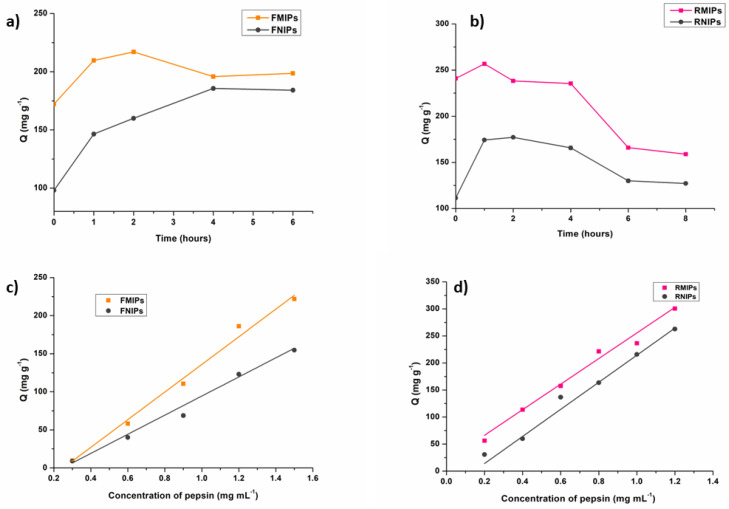
Binding kinetics for (**a**) FMIPs and FNIPs, and (**b**) RMIPs and RNIPs, and binding isotherm for (**c**) FMIPs and FNIPs, and (**d**) RMIPs and RNIPs.

The results of the binding kinetics experiments are graphically shown in Figure 6a,b. Here, we notice a difference between fluorescein-dyed polymers and rhodamine b-dyed polymers. We observe that the peak of binding for FMIPs takes place after 2 h; however, for RMIPs, maximum binding was achieved after only 1 h. We can attribute the faster binding kinetics of RMIPs to the presence of the two diethylamino groups in the structure of rhodamine b, in which their positive charge can interact significantly with the negatively charged pepsin molecule, resulting in faster binding kinetics. Moreover, the value of Q (mg g^−1^) at the maximum binding time of RMIPs (256) is higher than the corresponding Q value (mg g^−1^) at the maximum binding time for FMIPs (217), which again verifies the role played by the diethylamino groups in enhancing the binding of pepsin. In an attempt to recognise the mechanism of binding of pepsin to the developed polymers, pseudo-first-order and pseudo-second-order kinetics parameters were processed to fit the adsorption data. The data shown in Table 2 reflects that both rhodamine b and fluorescein imprinted polymers follow a pseudo-second-order model, which confirms that binding follows a chemical adsorption mechanism.

The graphical representation of the binding isotherm for both fluorescein and rhodamine b polymers (Figure 6c,d) shows a linear relationship between the binding capacity (Q) and the concentration of pepsin. The Freundlich isotherm and the Langmuir isotherm models were applied to the binding isotherm data, and the results are shown in Table 3. From the computed R^2^ values, we can assume that fluorescein-dyed polymers follow the Freundlich binding isotherm model, which suggests that the binding is in multiple layers on a heterogeneous surface. On the contrary, rhodamine b dyed polymers followed the Langmuir binding model, which indicates that binding occurs in a monolayer over a homogenous surface, which again proves the superiority of RMIPs.

A binding selectivity assay towards pepsin was conducted for both FMIPs versus FNIPs and for RMIPs versus RNIPs, and the imprinting factor (IF) was computed using the formula.

$$IF=\frac{Q_{MIPs\;}}{Q_{NIPs}}$$

Using a concentration of 1 mg mL^−1^ of pepsin, the IF values were 1.13 for fluorescein dyed polymers and 1.89 for rhodamine b dyed polymers, which proved the higher selectivity of RMIPs compared to FMIPs.

**Figure 7:**
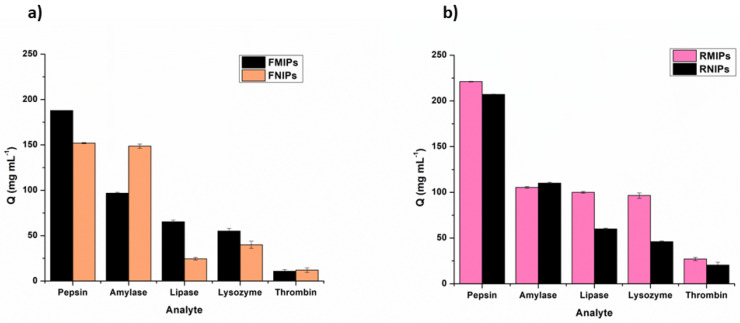
Selectivity studies of (**a**) FMIPs andFNIPs and (**b**) RMIPs, and RNIPs for pepsin against other proteins.

Furthermore, selectivity was tested against lipase and amylase due to their coexistence with pepsin in human saliva along with lysozyme and thrombin as other proteins. The results shown in Figure 7 show remarkable selectivity towards pepsin in comparison to other proteins for both fluorescein and rhodamine b dyed polymers, while again, RMIPs showed superior selectivity, indicated by the values of the separation factor (*α*) calculated using the formula.

$$\alpha =\frac{Q_{MIP\cdot target}}{Q_{MIP\cdot \;competitor}}$$

### 3.3. Stability Testing

**Figure 8:**
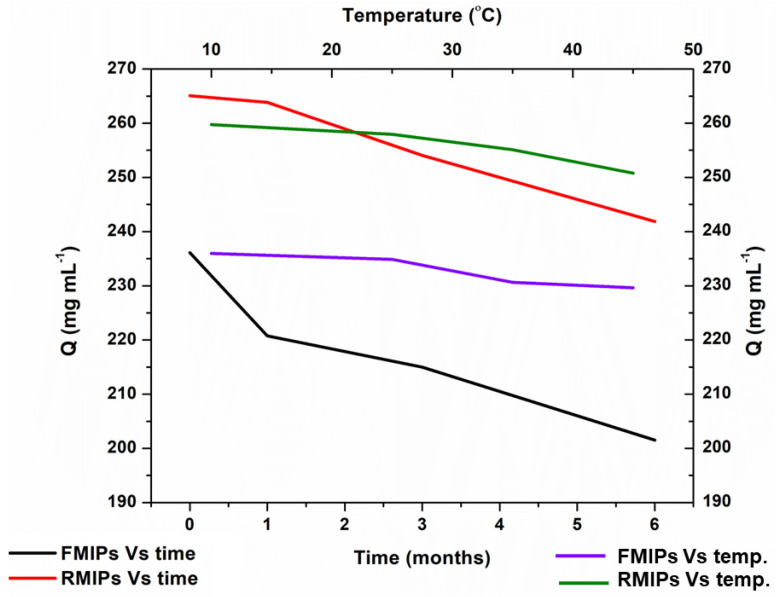
Stability of FMIPs and RMIPs against time and temperature.

The results of the stability testing conducted over the period of 0-, 1-, 3-, and 6- months showed a very slight decline in the binding capacity over the six month time period for both FMIPs and RMIPs. Similar results were obtained for testing at low and high temperatures, where the binding capacity for both FMIPs and RMIPs remained almost the same at all temperatures. Results are depicted collectively in Figure 8. These results provide proof of the superiority of MIPs to their corresponding antibodies in terms of physical stability.

### 3.4. Mechanism of Fluorescence Quenching

To investigate the mechanism of fluorescence quenching observed for FMIPs and RMIPs in the presence of pepsin, the Stern-Volmer equation was used.

$$\frac{F0}{F}=1+Ksv[Q]$$

**Figure 9:**
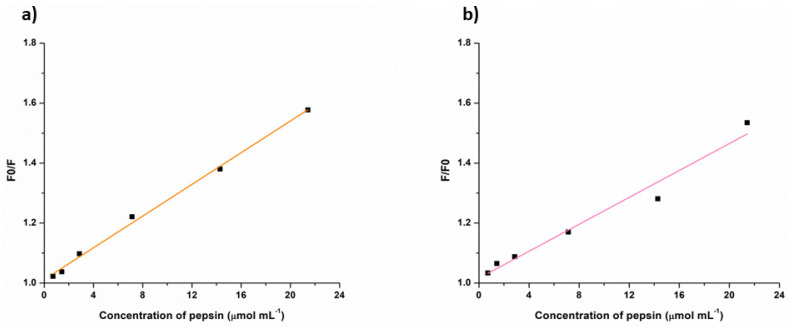
Graphical representation of Stern Volmer relationship between concentration of pepsin and the fluorescence quenching for (**a**) FMIPs and (**b**) RMIPs.

In which *F*0 is the fluorescence intensity of the fluorophore without the quencher, *F* is the fluorescence intensity after quenching, *Ksv* is the Stern-Volmer constant, and *Q* is the quencher concentration. Ideally, a linear relationship observed in the Stern-Volmer plot (*F*0/*F*) versus quencher concentration ([*Q*]) indicates the dominance of a single quenching process (dynamic quenching). Deviations from linearity in Stern-Volmer plots suggest the involvement of dual quenching mechanisms simultaneously, where dynamic quenching and static quenching play roles [35,36]. As noticed in Figure 9, a linear relationship between *F*0/*F* and the concentration of pepsin (the quencher) is observed for both FMIPs (Figure 9a) and RMIPs (Figure 9b). This linear relationship reflects a dynamic quenching of fluorescence in which the quencher interacts with the fluorophore at the excited state via collisions, leading to a return to the ground state through non-radiative pathways.

### 3.5. Quantitative Detection of Pepsin 

**Figure 10:**
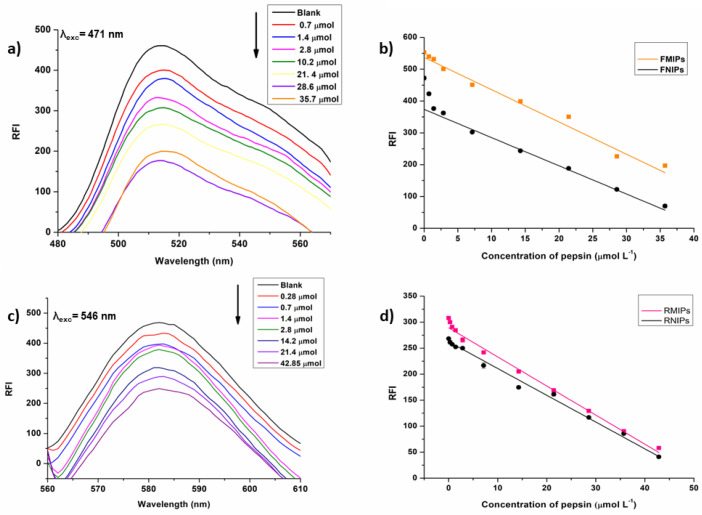
(**a**) Fluorescence measurements for FMIPs, (**b**) calibration curve for FMIPs and FNIPs, (**c**) fluorescence measurements for RMIPs, and (**d**) calibration curve for RMIPs and RNIPs.

The fluorescence intensity of both solutions of FMIPs and RMIPs was measured in triplicate at their respective excitation wavelengths (λ_exc_) after adding increased concentrations of pepsin from 0 (blank) to 42.85 µmol L^−1^. It was noted that the addition of pepsin caused a quenching in fluorescence intensity in a concentration-dependent fashion, as demonstrated in Figure 10. However, at higher concentrations, fluorescence response started to reach a plateau, which indicated the complete saturation of the binding sites for both FMIPs and RMIPs. The calculated linearity parameters for both MIPs are shown in Table 4. Interestingly, RMIPs showed a wider linear range extending to a lower concentration limit of 0.28 µmol L^−1^ and a higher limit of 42.85 µmol L^−1^ compared to FMIPs, whose lower limit started at 0.71 µmol L^−1,^ and the higher limit reached 35.71 µmol L^−1^. This reinforces the role of the structure of the fluorescent dye in the binding of the target and the sensitivity of the resulting MIPs. Therefore, RMIPs have more capability to bind pepsin even at lower concentrations compared to FMIPs resulting in superior sensitivity. In addition, the limit of detection value was computed for both FMIPs and RMIPs using the following equation:

$$LOD=\frac{3.3\cdot \delta }{slope}$$

where *δ* is the standard error of the intercept of the regression equation. 

The computed LOD values further confirmed our hypothesis. Where LOD for FMIPs is 0.36 µmol L^−1^, while the value reached 0.12 µmol L^−1^ for RMIPs. In our study, we have achieved an LOD of 0.12 µmol L^−1^ for pepsin using our novel fluorescent RMIPs. While direct comparison with Peptest™ in terms of concentration units may not be straightforward due to differences in assay methodologies, our MIP-based approach offers a comparable sensitivity to conventional diagnostic tests for pepsin detection [25]. Furthermore, our MIPs offer several advantages over traditional methods, including enhanced selectivity, ease of use, and potential for integration into point-of-care diagnostic devices. These attributes make our MIPs a promising tool for sensitive and specific detection of pepsin in various biological samples, including saliva.

### 3.6. Development of a Separation-Free Quantitation System for Pepsin in Human Saliva Samples

The fluorescent MIPs were effectively applied to quantify pepsin in human saliva samples. Furthermore, due to the high selectivity of the developed FMIPs and RMIPs, there was no need for a prior extraction step, therefore eliminating an extra step in sample preparation and allowing for a sensitive, simple, and one-step assay. Linearity in saliva ranged from 1.42 to 42.85 µmol L^−1^ for RMIPs and from 2.8 to 35.71 µmol L^−1^ for FMIPs. In addition, the lower concentrations measured by both the developed MIPs are very suitable for measuring the very low concentrations of pepsin found in the saliva of real GERD patients. Moreover, the percentage of recovery was calculated for pepsin from three different spiked samples containing three different concentrations along the calibration range. Percentages of recovery ranged from 94.8 to 101.2% for FMIPs and from 96.29 to 100.21% for RMIPs; results are demonstrated in Table 5.

## 4. Conclusions

In this work, we investigated the effect of the structure of the fluorescent dye on the selectivity and sensitivity of fluorescent molecularly imprinted polymers. Two fluorescent MIPs were prepared for the pepsin enzyme using two fluorescent dyes: fluorescein and rhodamine b. Comparing the performance of both MIPs in terms of binding capacity, binding selectivity, and quantitation range of pepsin confirmed the assumption that rhodamine b can offer superior results owing to establishing two extra bonds with pepsin in comparison to fluorescein. Thus, rhodamine b is assumed to provide three points of interaction with the pepsin molecule. However, fluorescein provides only one point of interaction. This highlights the importance of applying a structure-based approach in the choice of fluorescent dyes and monomers for the preparation of fluorescent MIPs. Furthermore, the developed FMIPs and RMIPs were both applied successfully for the detection and quantitation of pepsin in solutions and saliva samples, with RMIPs achieving higher sensitivity, indicated by a LOD value of 0.12 µmol L^−1^ compared to FMIPs with a LOD value of 0.36 µmol L^−1^. Therefore, both fluorescent MIPs provided the advantage of an integrated extraction and analysis tool in comparison to the magnetic MIPs our group previously developed, which acted as extraction tools only. 

## Figures and Tables

**Table 1:** ANOVA parameters for testing the effect of particle size variability on the reproducibility of the fluorescence signal for FMIPs, FNIPs, RMIPs, and RNIPs.

Polymer	SSB	SSW	SST	F Calculated	F Critical (α = 0.05)
FMIPs	47.195	180.125	227.321	0.611	2.847
FNIPs	3.619	13.163	16.782	0.6415	2.847
RMIPs	7.272	24.678	31.950	0.687	2.847
RNIPs	6.404	60.741	67.146	0.246	2.847

SSB: sum of squares between groups, SSW: sum of squares within groups, SST: sum of squares for total data, F calculated: is the ratio of the mean square between groups to the mean square within groups, F critical: s the critical value of the F-distribution for the chosen significance level (α = 0.05).

**Table 2:** Binding kinetics parameters for the adsorption of pepsin applying for pseudo-first and pseudo-second binding orders.

**Pseudo First Order Parameters**					
**FMIPs**			**FNIPs**		
K_1_ (min^−1^)	Qe (mg g^−1^)	R^2^	K_1_ (min^−1^)	Qe (mg g^−1^)	R^2^
0.00077	25.432	0.0009	0.0309	81.183	0.872
**RMIPs**			**RNIPs**		
K_1_ (min^−1^)	Qe (mg g^−1^)	R^2^	K_1_ (min^−1^)	Qe (mg g^−1^)	R^2^
0.033	8.11	0.66	0.0173	10.175	0.163
**Pseudo Second Order Parameters**					
**FMIPs**			**FNIPs**		
K_2_ (g mg^−1^ min^−1^)	Qe (mg g^−1^)	R^2^	K_2_ (g mg^−1^ min^−1^)	Qe (mg g^−1^)	R^2^
0.0867	196.09	0.9989	0.02809	188.68	0.9961
**RMIPs**			**RNIPs**		
K_2_ (g mg^−1^ min^−1^)	Qe (mg g^−1^)	R^2^	K_2_ (g mg^−1^ min^−1^)	Qe (mg g^−1^)	R^2^
0.0136	156.25	0.9866	0.022	125	0.9759

K_1_ and K_2_ are the first and second order rate constants respectively, Qe is the quantity of pepsin adsorbed per gram of polymer at equilibrium, and R^2^ is the linearity coefficient.

**Table 3:** Adsorption isotherm parameters of MIPs and NIPs applying two models.

**Langmuir Isotherm**							
**FMIPs**				**FNIPs**			
K_L_ (L mg^−1^)	Q_max_ (mg g^−1^)	RL	R^2^	K_L_ (L mg^−1^)	Q_max_ (mg g^−1^)	RL	R^2^
0.002	19.45	1.002	0.9055	0.00158	25.22	1.001	0.9460
**RMIPs**				**RNIPs**			
K_L_ (L mg^−1^)	Q_max_ (mg g^−1^)	RL	R^2^	K_L_ (L mg^−1^)	Q_max_ (mg g^−1^)	RL	R^2^
1.241	830.67	0.573	0.9887	1.226	174.093	3.782	0.9360
**Freundlich Isotherm**							
**FMIPs**				**FNIPs**			
n	K_F_		R^2^	n	K_F_		R^2^
0.381	349.869		0.9599	0.470	156.50		0.9428
**RMIPs**				**RNIPs**			
n	K_F_		R^2^	n	K_F_		R^2^
1.285	553.384		0.9615	0.683	658.99		0.9117

K_L_ and K_F_ are the Langmuir constant and Freundlich constant, respectively. Q_max_ is the theoretical maximum adsorbed concentration, RL is the separation factor (1/1 + C_eq_·KL), n is the variation trend coefficient for the adsorption isotherm, and R^2^ is the linearity coefficient.

**Table 4:** Linearity parameters for quantitation of pepsin in standard solutions using FMIPs and RMIPs.

Parameter	Linearity Range (µmol L^−1^)	Intercept ± SD ^a^	Slope ± SD ^b^	S_yx_^c^	R^2 d^	LOD (µmol L^−1^) ^e^
FMIPs	0.71–35.71	551.94 ±1.61	−0.42 ± 0.050	1.769	0.9842	0.36 ± 0.051
RMIPs	0.28–42.85	307.53 ± 0.85	−0.67 ± 0.055	0.9733	0.9916	0.12 ± 0.048

^a^ Standard deviation of the intercept, ^b^ Standard deviation of the slope, ^c^ Sum of square errors, ^d^ R the correlation coefficient, ^e^ LOD limit of detection.

**Table 5:** Recovery of spiked pepsin from saliva samples.

Concentration (µmol mL^−1^)	Average Total the Amount Found (µmol mL^−1^)		Average% Recovery ± SD		RSD %	
	FMIPs	RMIPs	FMIPs	RMIPs	FMIPs	RMIPs
2.8	2.65	2.69	94.84 ± 3.39	96.07 ± 3.07	3.57	3.19
14.28	14.24	14.31	99.76 ± 1.51	100.21 ± 2.08	1.52	2.08
35.71	36.14	35.50	101.22 ± 0.312	99.42 ± 0.99	0.31	1.00

SD is the standard deviation, and RSD is the relative standard deviation.

## Data Availability

All data generated or analysed during this study are included in this published article and its Supplementary Materials. Any additional data will be available upon request.

## Supplementary Materials

The following supporting information can be downloaded at: https://www.mdpi.com/article/10.3390/bios14030151/s1, Figure S1. Pictures of the developed FMIPs, FNIPs, RMIPs, and RNIPs on the bench and under long-wave UV light. Figure S2. Results of different optimisation experiments for FMIPs and their corresponding FNIPs. Figure S3. Results of different optimisation experiments for RMIPs and their corresponding RNIPs. Figure S4. ^13^C NMR spectra of FITC and FITC-APTES. Figure S5. ^13^C NMR spectra of RITC and RITC-APTES.

## References

[1] Haupt K., Mosbach K. (2000). Molecularly Imprinted Polymers and Their Use in Biomimetic Sensors. Chem. Rev..

[2] Vasapollo G., Del Sole R., Mergola L., Lazzoi M.R., Scardino A., Scorrano S., Mele G. (2011). Molecularly Imprinted Polymers: Present and Future Prospective. Int. J. Mol. Sci..

[3] Quinn T. (2016). Molecularly Imprinted Polymers (MIPS) Challenges, Uses and Prospects.

[4] Arabi M., Ostovan A., Li J., Wang X., Zhang Z., Choo J., Chen L. (2021). Molecular Imprinting: Green Perspectives and Strategies. Adv. Mater..

[5] Mostafa A.M., Barton S.J., Wren S.P., Barker J. (2021). Review on Molecularly Imprinted Polymers with a Focus on Their Application to the Analysis of Protein Biomarkers. TrAC Trends Anal. Chem..

[6] Parisi O.I., Francomano F., Dattilo M., Patitucci F., Prete S., Amone F., Puoci F. (2022). The Evolution of Molecular Recognition: From Antibodies to Molecularly Imprinted Polymers (MIPs) as Artificial Counterpart. J. Funct. Biomater..

[7] Zhao Y., Chen Y., Fang M., Tian Y., Bai G., Zhuo K. (2020). Silanized Carbon Dot-Based Thermo-Sensitive Molecularly Imprinted Fluorescent Sensor for Bovine Hemoglobin Detection. Anal. Bioanal. Chem..

[8] Fang M., Zhuo K., Chen Y., Zhao Y., Bai G., Wang J. (2019). Fluorescent Probe Based on Carbon Dots/Silica/Molecularly Imprinted Polymer for Lysozyme Detection and Cell Imaging. Anal. Bioanal. Chem..

[9] Piloto A.M.L., Ribeiro D.S.M., Rodrigues S.S.M., Santos J.L.M., Ferreira Sales M.G. (2020). Label-Free Quantum Dot Conjugates for Human Protein IL-2 Based on Molecularly Imprinted Polymers. Sens. Actuators B Chem..

[10] Zhang X., Yang S., Sun L., Luo A. (2016). Surface-Imprinted Polymer Coating l-Cysteine-Capped ZnS Quantum Dots for Target Protein Specific Recognition. J. Mater. Sci..

[11] Zhou T., Halder A., Sun Y. (2018). Fluorescent Nanosensor Based on Molecularly Imprinted Polymers Coated on Graphene Quantum Dots for Fast Detection of Antibiotics. Biosensors.

[12] Piloto A.M., Ribeiro D.S.M., Rodrigues S.S.M., Santos C., Santos J.L.M., Sales M.G.F. (2018). Plastic Antibodies Tailored on Quantum Dots for an Optical Detection of Myoglobin down to the Femtomolar Range. Sci. Rep..

[13] Pirzada M., Sehit E., Altintas Z. (2020). Cancer Biomarker Detection in Human Serum Samples Using Nanoparticle Decorated Epitope-Mediated Hybrid MIP. Biosens. Bioelectron..

[14] Feng H., Wang N., Yuan L., Li J., Cai Q. (2014). Surface Molecular Imprinting on Dye-(NH_2_)-SiO_2_ NPs for Specific Recognition and Direct Fluorescent Quantification of Perfluorooctane Sulfonate. Sens. Actuators B Chem..

[15] Wan W., Biyikal M., Wagner R., Sellergren B., Rurack K. (2013). Fluorescent Sensory Microparticles That “Light-up” Consisting of a Silica Core and a Molecularly Imprinted Polymer (MIP) Shell. Angew. Chem. Int. Ed..

[16] Xu Z., Deng P., Tang S., Li J. (2016). Fluorescent Molecularly Imprinted Polymers Based on 1,8-Naphthalimide Derivatives for Efficiently Recognition of Cholic Acid. Mater. Sci. Eng. C.

[17] Sunayama H., Ohta T., Kuwahara A., Takeuchi T. (2016). Fluorescence Signaling Molecularly Imprinted Polymers for Antibiotics Prepared via Site-Directed Post-Imprinting Introduction of Plural Fluorescent Reporters within the Recognition Cavity. J. Mater. Chem. B.

[18] Wan W., Wagner S., Rurack K. (2016). Fluorescent Monomers: “Bricks” That Make a Molecularly Imprinted Polymer “Bright”. Anal. Bioanal. Chem..

[19] Chen L., Xu S., Li J. (2011). Recent Advances in Molecular Imprinting Technology: Current Status, Challenges and Highlighted Applications. Chem. Soc. Rev..

[20] Liu R., Ko C.C. (2023). Molecularly Imprinted Polymer-Based Luminescent Chemosensors. Biosensors.

[21] Wang F., Wang D., Wang T., Jin Y., Ling B., Li Q., Li J. (2021). A Simple Approach to Prepare Fluorescent Molecularly Imprinted Nanoparticles. RSC Adv..

[22] Boulton K.H.A., Dettmar P.W. (2022). A Narrative Review of the Prevalence of Gastroesophageal Reflux Disease (GERD). Ann. Esophagus.

[23] Samloff I.M., Taggart R.T. (1987). Pepsinogens, Pepsins, and Peptic Ulcer. Clin. Investig. Med..

[24] Wang Y.J., Lang X.Q., Wu D., He Y.Q., Lan C.H., Xiao X., Wang B., Zou D.W., Wu J.M., Zhao Y.B. (2020). Salivary Pepsin as an Intrinsic Marker for Diagnosis of Sub-Types of Gastroesophageal Reflux Disease and Gastroesophageal Reflux Disease-Related Disorders. J. Neurogastroenterol. Motil..

[25] Zeleník K., Hránková V., Vrtková A., Staníková L., Komínek P., Formánek M. (2021). Diagnostic Value of the Peptest^TM^ in Detecting Laryngopharyngeal Reflux. J. Clin. Med..

[26] Verheyen E., Schillemans J.P., Van Wijk M., Demeniex M., Hennink W.E., Van Nostrum C.F. (2011). Challenges for the Effective Molecular Imprinting of Proteins. Biomaterials.

[27] Ansari S., Masoum S. (2019). Molecularly Imprinted Polymers for Capturing and Sensing Proteins: Current Progress and Future Implications. TrAC Trends Anal. Chem..

[28] Pluhar B., Ziener U., Mizaikoff B. (2015). Binding Performance of Pepsin Surface-Imprinted Polymer Particles in Protein Mixtures. J. Mater. Chem. B.

[29] Piletska E.V., Czulak J., Piletsky S.S., Guerreiro A., Canfarotta F., Piletsky S.A. (2018). Novel Assay Format for Proteins Based on Magnetic Molecularly Imprinted Polymer Nanoparticles—Detection of Pepsin. J. Chin. Adv. Mater. Soc..

[30] García Y., Czulak J., Pereira E.D., Piletsky S.A., Piletska E. (2022). A Magnetic Molecularly Imprinted Nanoparticle Assay (MINA) for Detection of Pepsin. React. Funct. Polym..

[31] Mostafa A.M., Barton S.J., Wren S.P., Barker J. (2022). Development of Magnetic Molecularly Imprinted Polymers for the Extraction of Salivary Pepsin Prior to Analysis by a Novel HPLC-SEC Method. Polymer.

[32] Wang S., Yin D., Wang W., Shen X., Zhu J.J., Chen H.Y., Liu Z. (2016). Targeting and Imaging of Cancer Cells via Monosaccharide-Imprinted Fluorescent Nanoparticles. Sci. Rep..

[33] Baliga S., Muglikar S., Kale R. (2013). Salivary pH: A Diagnostic Biomarker. J. Indian Soc. Periodontol..

[34] Fenger F., Andrew R.H. (1927). On the Isoelectric Precipitation of Pepsin. J. Biol. Chem..

[35] Paterson K.A., Arlt J., Jones A.C. (2020). Dynamic and Static Quenching of 2-Aminopurine Fluorescence by the Natural DNA Nucleotides in Solution. Methods Appl. Fluoresc..

[36] Gentleman A.S., Lawson T., Ellis M.G., Davis M., Turner-Dore J., Ryder A.S.H., Frosz M.H., Ciaccia M., Reisner E., Cresswell A.J. (2022). Stern-Volmer Analysis of Photocatalyst Fluorescence Quenching within Hollow-Core Photonic Crystal Fibre Microreactors. Chem. Commun..

