# Supplementary material for: Development of Highly Sensitive Fluorescent Sensors for Separation-Free Detection and Quantitation Systems of Pepsin Enzyme Applying a Structure-Guided Approach
Source: Biosensors (Basel). 2024 Mar 20;14(3):151. doi: 10.3390/bios14030151 (PMC10968241; doi:10.3390/bios14030151)
Supplement: Supplementary file 1 [file biosensors-14-00151-s001.zip › biosensors-2928482-supplementary.pdf]

Electronic Supplementary Material

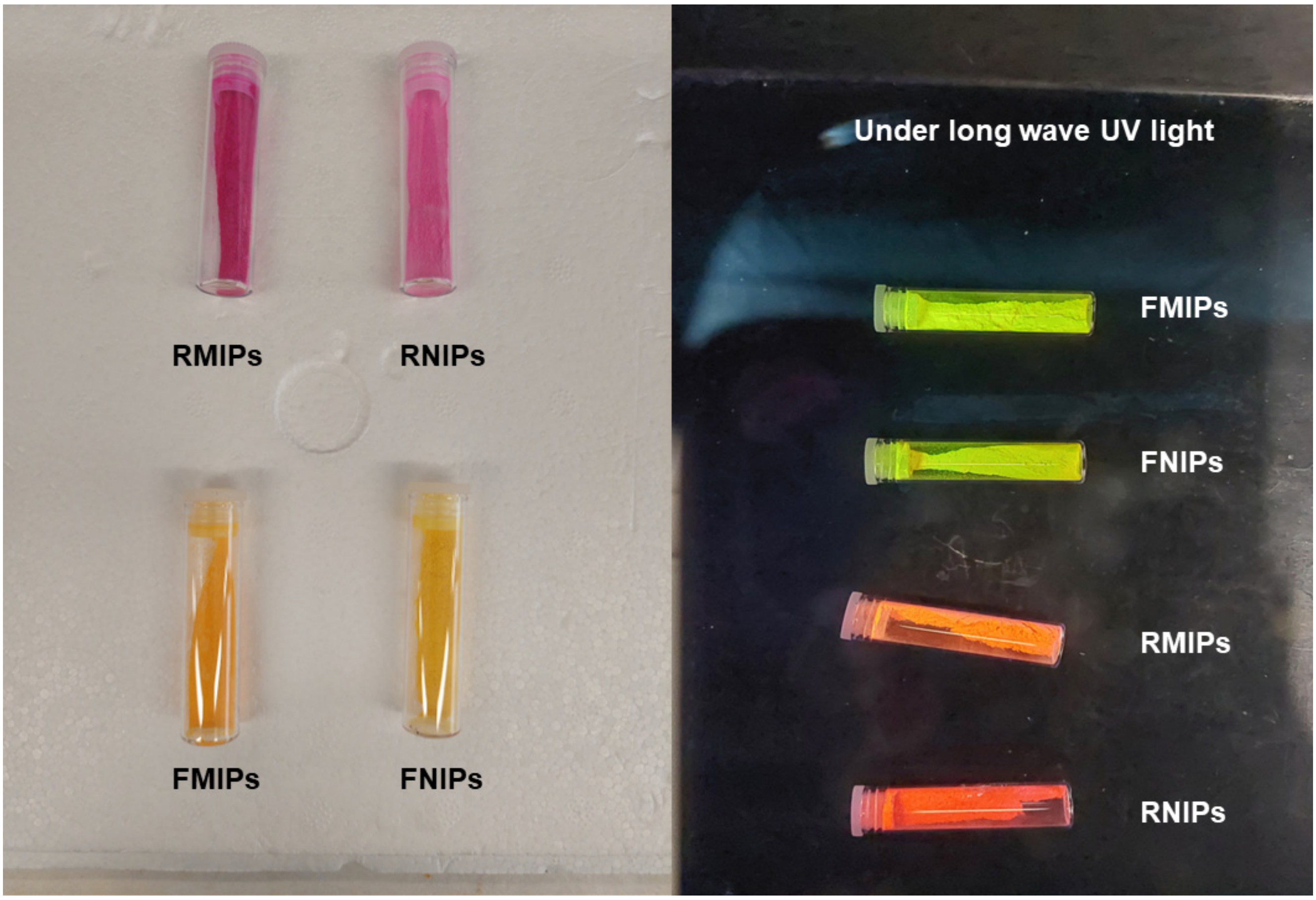

Figure S1. Pictures of the developed FMIPs, FNIPs, RMIPs, and RNIPs on the bench and under long wave UV light.

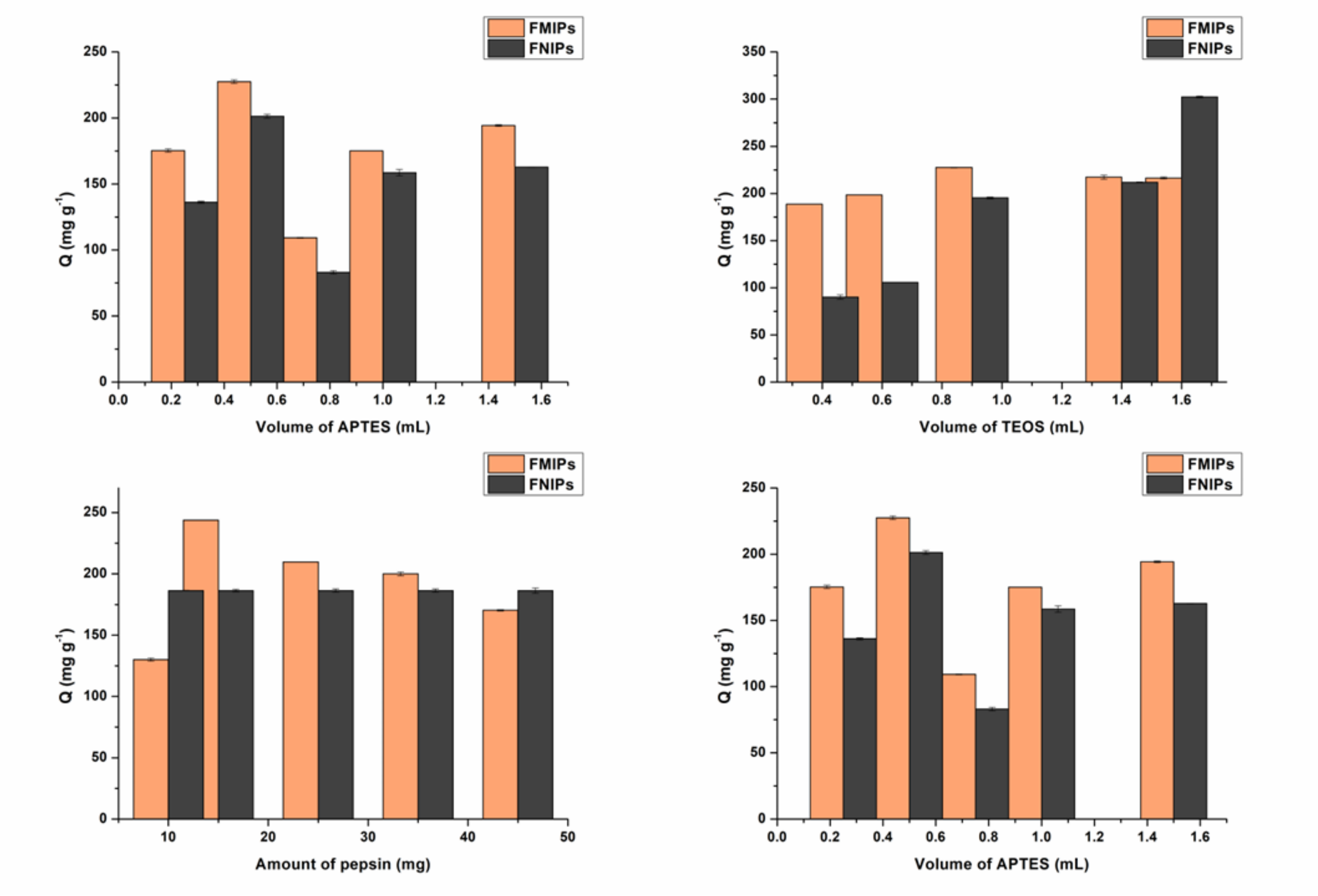

Figure S2. Results of different optimisation experiments for FMIPs and their corresponding FNIPs.

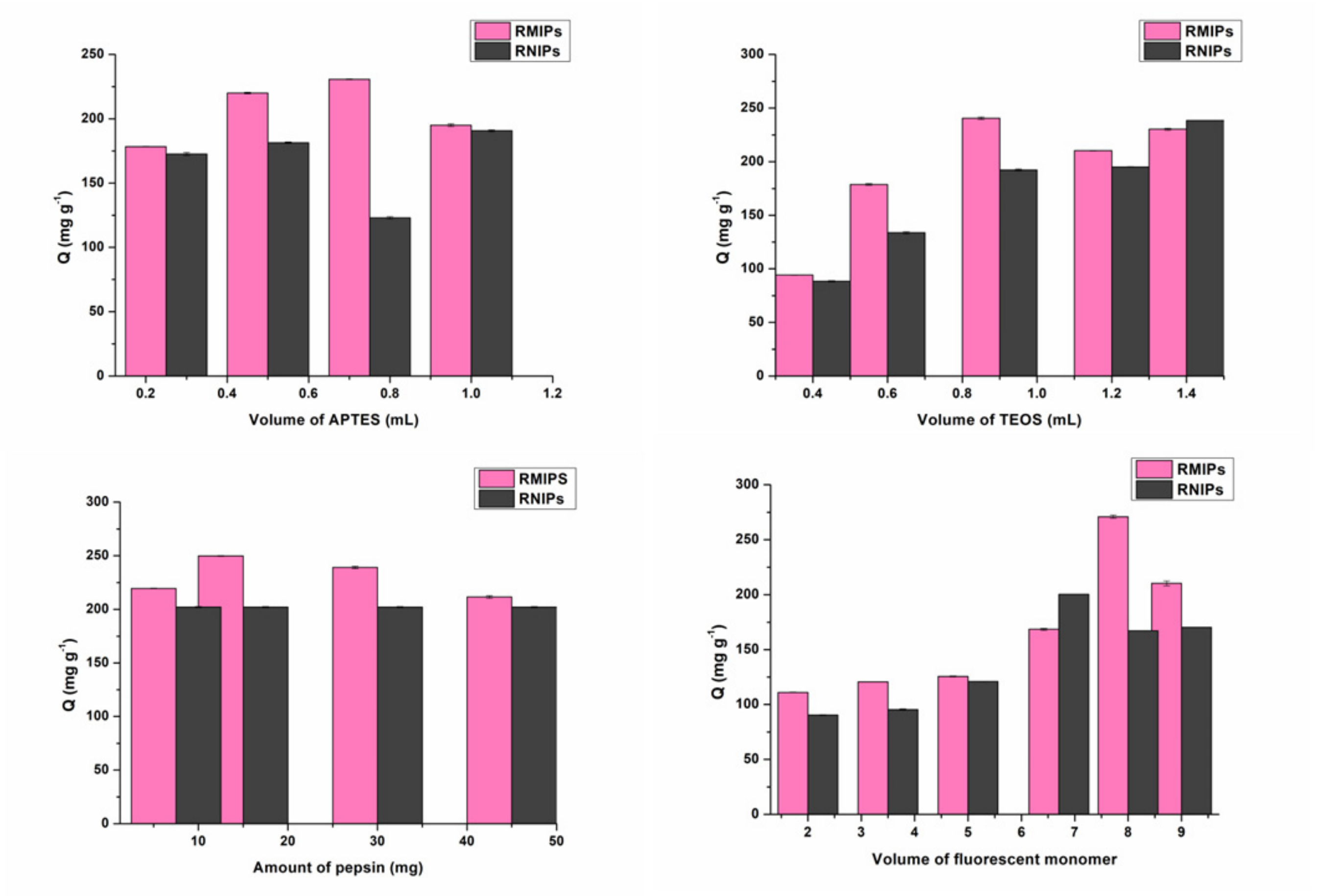

Figure S3. Results of different optimisation experiments for RMIPs and their corresponding RNIPs.

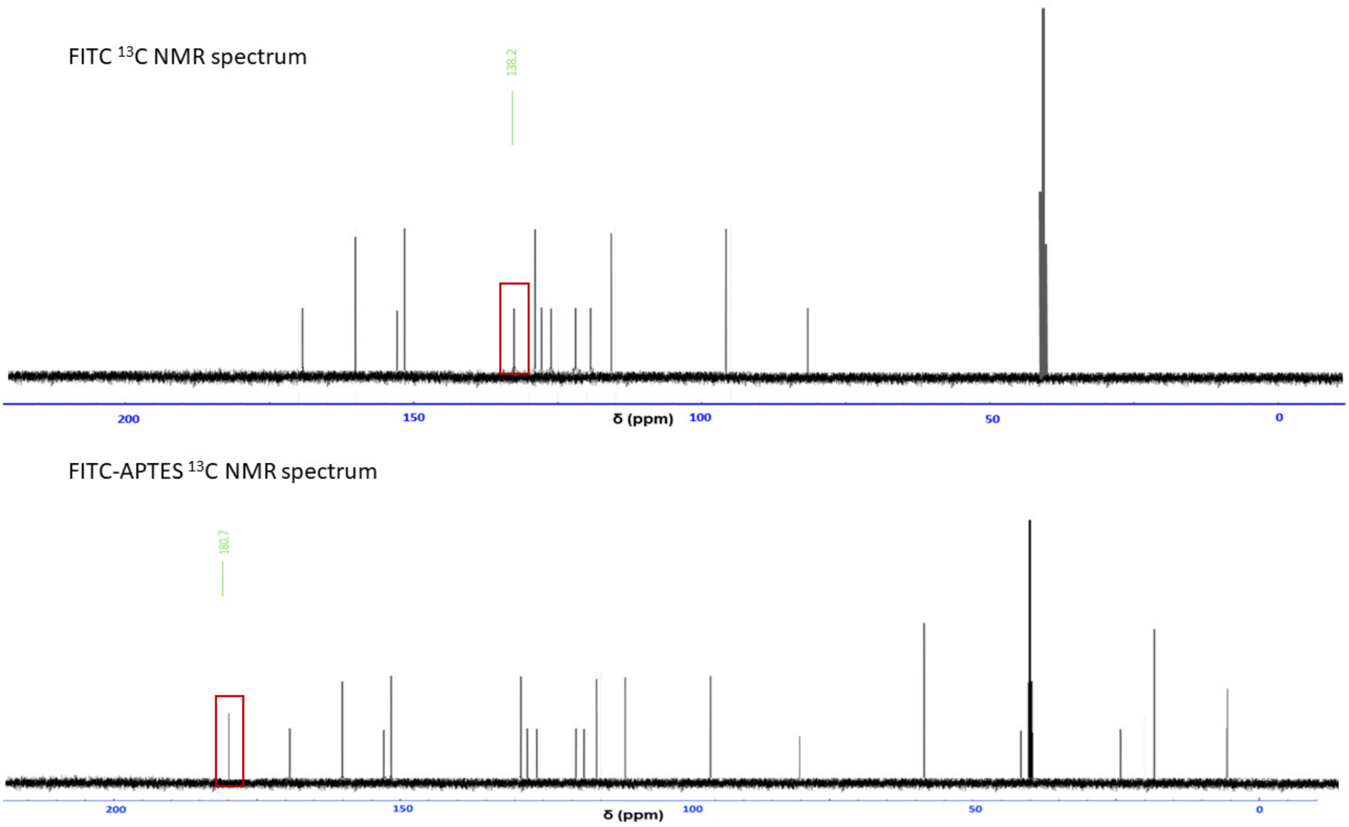

Figure S4. ^{13}C NMR spectra of FITC and FITC-APTES

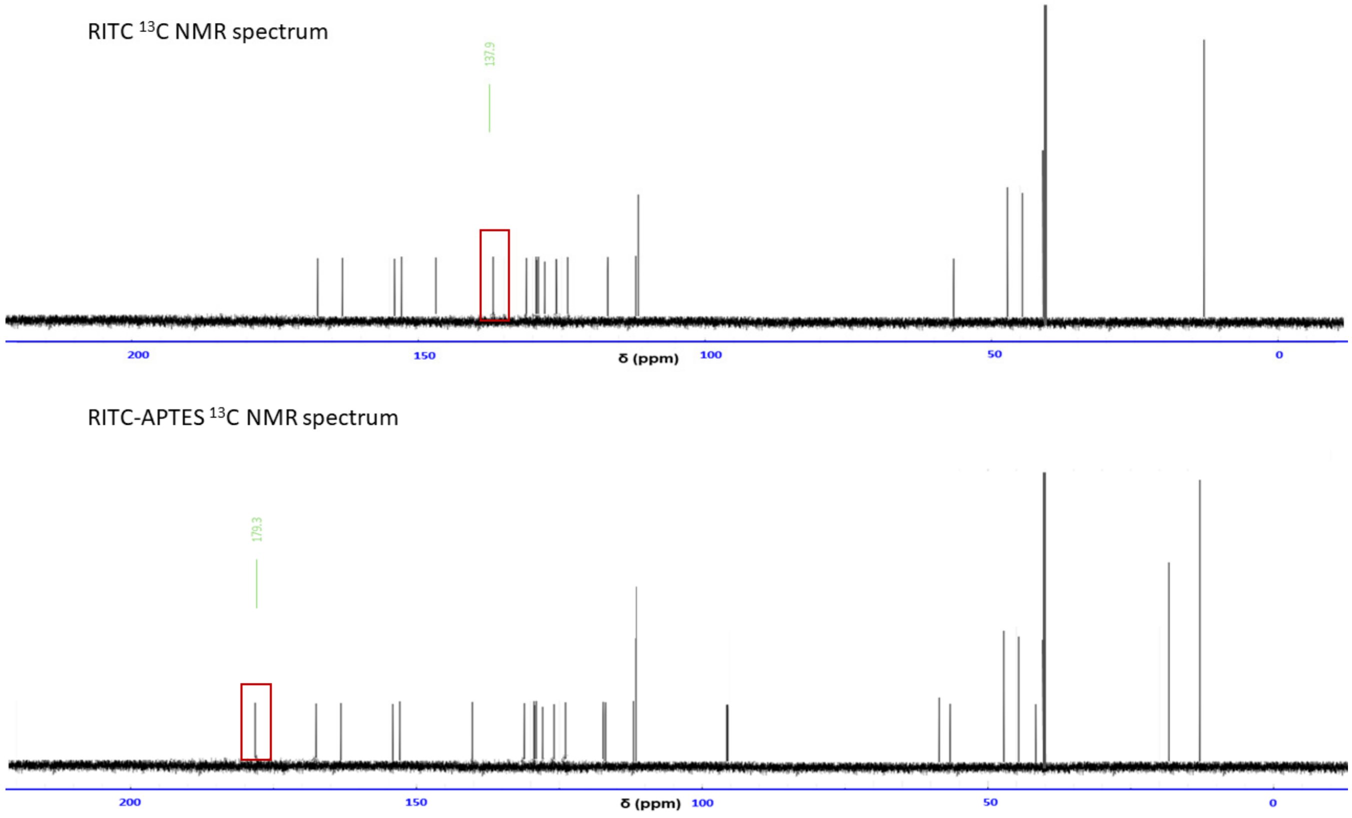

Figure S5. ^{13}C NMR spectra of RITC and RITC-APTES
